# Use of Toilet Soap

**Published:** 1887-11

**Authors:** Hester M. Poole


### USE OF TOILET SOAP.

The opinion that of such a necessary article as soap for the toilet one can't use too much, is an opinion which late researches in science disprove. The attraction of the alkali in it for the oil of the skin as well as for its unclean accumulation, constitutes its cleansing property. Out of the 7,000,000 pores through which nearly two pounds of poisonous exhalations daily pass from the adult, come enough materials in a short time to produce fatal and filthy diseases. An eminent physician has declared that "if the skin be moderately active, three or four days suffice to form a layer which may be compared to a thin coating of varnish or sizing." As this accumulation increases and decomposition follows it is not necessary to describe the result. What agency but soap can remove it ?

Many good authorities declare that water alone is sufficient, except at rare intervals. There are oil glands as well as excretory ducts, and for no idle purpose has nature produced these tiny human oil wells. Inunction, or the external use of oil, has a recognized place among the prescriptions of some famous modern physicians, who in this way seek to restore that necessary property of which the body has been deprived, by the excessive use of soap or by disease. They claim that it enables the patient to resist cold, that its nutritive qualities convey heat to those organs which require it, that it gives a sense of exhilarating freshness, and that it is not only soothing in cases of nervous depression, but it is capable of strengthening weak lungs. For this purpose almond oil, cocoanut, olive oil or vaseline are daily applied by the aid of vigorous rubbing. To all such treatment, and in most cases where inunction is not required, the daily application of soaps is injurious.

"What uncleanly habits!" some one exclaims. Not so. Plenty of soft water, a coarse wash rag, hand friction and a Turkish towel, with soap applied at rare intervals, and the skin should retain the delicate smoothness of an infant. Those milk baths indulged in by the ancient Roman emperors and empresses owed their emollient properties to the oil contained in the milk. Every old nurse knows, too, that weakly children are sometimes injured by too frequent ablutions. Dry rubbing is often the safest opiate for a nervous little one, answering many of the purposes of soap.

An eminent physician and scientist lately told me that he seldom used soap in his daily bath. "It makes the skin dry, hard and harsh, and renders me much more liable to take cold through any changes of the weather," said he. "At the same time, no rule can be given for the soap. Some persons secrete oil much more readily than others, and to such soap is more of a necessity," and he spoke much upon the desirability of using a pure soap or none at all.—*Hester M. Poole, in Good Housekeeping.*

